# Queen's Jubilee Hospital

**Published:** 1899-02-11

**Authors:** 


### THE INSTITUTIONAL WORKSHOP.

### QUEEN'S JUBILEE HOSPITAL.

### THE MATRON DIAGNOSES THE CASES.

WE publish the following from the *Pall Mall Gazette* of February 6th:—

The West London coroner, Mr. C. L. Drew, held an inquiry at the Kensington Town Hall on Saturday with reference to the death of Henry Goldsmith, a cement merchant's carman. The deceased was driving on Tuesday morning in Fulham Road, when he suddenly pitched from his van on his head in the roadway. Police-constable Benjamin Thompson said he took the deceased to the Queen's Jubilee Hospital, and the matron said he was dead. There was no doctor there then, but the doctor came about twenty minutes after.

Sophia Smith, the matron of the hospital, said the man was dead on admission. She at once sent for the house surgeon, who lived at West Kensington.

How long does it take to fetch him?—About a quarter of an hour by cab.

Suppose he is not at home?—Dr. Benham lives close to him.

In answer to other questions, the witness said they had eight beds in the hospital, and that the house surgeon was there nearly all day. If the doctors were not at home she would, if necessary, call in the nearest outside doctor.

The Coroner said it caused a very serious delay. He asked the matron if a child were brought in suffering from spasm of throat and tracheotomy had to be performed at once what she would do. The witness replied she should send for a doctor at once.

Do you call a doctor in every case?—No; only in urgent cases.

Then it is practically left to you to diagnose a case and decide whether it is urgent or not.

Mr. Joseph Carbery, of 13, Pelham-road, West Kensington, a retired assistant colonial surgeon, said he had been honorary surgeon to the Jubilee Hospital for five months. He spent most of the day there. He could reach the hospital from his house by omnibus in ten minutes. He was called by a messenger on Tuesday and went at once. The man had been dead some time. He had made a post-mortem examination, and found that the skull had been almost split in two by the fall, and that this had been caused by a sudden attack of syncope. He found all the organs except the brain congested. This was recent. There was no food in the stomach.

The Coroner asked if the congestion of the organs did not indicate that the man had been a long while dying? The witness considered it was due to the cold.

The Coroner said the doctor must see the serious state of affairs. Here was a hospital with eight beds, and accidents coming in day and night, and yet it took twenty minutes to get a doctor. Who was to decide if a case was urgent? Dr. Carbery said there was a younger surgeon than himself as well.

The Coroner: Are you called to slight cases?—No, sir; they are dressed by the matron and sent away.

Does she diagnose whether they are slight or not?—The matron is an experienced person, and uses her common sense.

It was a perfectly plain question. Does the matron decide whether a case is an urgent one or not?—Yes.

Supposing it was a case of secondary hæmorrhage, what would happen?—The nurse would know to apply a tourniquet.

The jury returned a verdict of accidental death.

